# Combination effects of amino acid transporter LAT1 inhibitor nanvuranlat and cytotoxic anticancer drug gemcitabine on pancreatic and biliary tract cancer cells

**DOI:** 10.1186/s12935-023-02957-z

**Published:** 2023-06-15

**Authors:** Kou Nishikubo, Ryuichi Ohgaki, Xingming Liu, Hiroki Okanishi, Minhui Xu, Hitoshi Endou, Yoshikatsu Kanai

**Affiliations:** 1 Department of Bio-system Pharmacology, Graduate School of Medicine, Osaka University, 2-2 Yamadaoka, Suita, Osaka 565-0871 Japan; 2 Integrated Frontier Research for Medical Science Division, Institute for Open and Transdisciplinary Research Initiatives (OTRI), Osaka University, Osaka, Japan; 3 J-Pharma Co., Ltd., Yokohama, Japan

**Keywords:** Amino acid transporter, LAT1, SLC7A5, Large neutral amino acids, Essential amino acids, Molecularly targeted drugs, Cancer chemotherapy, Combination therapy, Cytotoxic anticancer drugs, Gemcitabine

## Abstract

**Background:**

Cytotoxic anticancer drugs widely used in cancer chemotherapy have some limitations, such as the development of side effects and drug resistance. Furthermore, monotherapy is often less effective against heterogeneous cancer tissues. Combination therapies of cytotoxic anticancer drugs with molecularly targeted drugs have been pursued to solve such fundamental problems. Nanvuranlat (JPH203 or KYT-0353), an inhibitor for L-type amino acid transporter 1 (LAT1; SLC7A5), has novel mechanisms of action to suppress the cancer cell proliferation and tumor growth by inhibiting the transport of large neutral amino acids into cancer cells. This study investigated the potential of the combined use of nanvuranlat and cytotoxic anticancer drugs.

**Methods:**

The combination effects of cytotoxic anticancer drugs and nanvuranlat on cell growth were examined by a water-soluble tetrazolium salt assay in two-dimensional cultures of pancreatic and biliary tract cancer cell lines. To elucidate the pharmacological mechanisms underlying the combination of gemcitabine and nanvuranlat, we investigated apoptotic cell death and cell cycle by flow cytometry. The phosphorylation levels of amino acid-related signaling pathways were analyzed by Western blot. Furthermore, growth inhibition was examined in cancer cell spheroids.

**Results:**

All the tested seven types of cytotoxic anticancer drugs combined with nanvuranlat significantly inhibited the cell growth of pancreatic cancer MIA PaCa-2 cells compared to their single treatment. Among them, the combined effects of gemcitabine and nanvuranlat were relatively high and confirmed in multiple pancreatic and biliary tract cell lines in two-dimensional cultures. The growth inhibitory effects were suggested to be additive but not synergistic under the tested conditions. Gemcitabine generally induced cell cycle arrest at the S phase and apoptotic cell death, while nanvuranlat induced cell cycle arrest at the G0/G1 phase and affected amino acid-related mTORC1 and GAAC signaling pathways. In combination, each anticancer drug basically exerted its own pharmacological activities, although gemcitabine more strongly influenced the cell cycle than nanvuranlat. The combination effects of growth inhibition were also verified in cancer cell spheroids.

**Conclusions:**

Our study demonstrates the potential of first-in-class LAT1 inhibitor nanvuranlat as a concomitant drug with cytotoxic anticancer drugs, especially gemcitabine, on pancreatic and biliary tract cancers.

**Supplementary Information:**

The online version contains supplementary material available at 10.1186/s12935-023-02957-z.

## Background

Conventional cytotoxic anticancer drugs are commonly used in current standard cancer chemotherapies. However, the development of adverse effects is inherently difficult to avoid in their clinical use [1, 2]. As they target nucleic acids or proteins involved in nucleic acid synthesis, DNA replication, transcription, and cell division, cytotoxic anticancer drugs inevitably damage normal proliferating cells besides cancer cells. In addition, drug resistance and tumor heterogeneity often limit the efficacy of monochemotherapies [3]. More effective therapeutic strategies have been continuously pursued to overcome such limitations. Those include the combined use of multiple cytotoxic anticancer drugs or cytotoxic anticancer drugs with molecularly targeted drugs.

Pancreatic and biliary tract cancers are the most aggressive malignancies with poor prognoses [4–6]. Due to the asymptomatic nature of the diseases at early stages, most patients are diagnosed at advanced stages that are not eligible for surgical resection. Gemcitabine (GEM), classified as an antimetabolite, is commonly used for drug treatments of these cancers [4–6]. The first-line therapies for unresectable or metastatic diseases include GEM-based combined therapies, i.e., GEM with nab-paclitaxel or erlotinib for pancreatic cancer [4, 5, 7, 8] and GEM with cisplatin or S-1 (tegafur, gimeracil, and oteracil potassium), or both, for biliary tract cancer [6, 9, 10]. However, these current therapies often develop dose-limiting myelosuppression (such as leukopenia, neutropenia, and thrombopenia), achieving only modest life-prolonging effects [4–6].

Cancer cells exhibit an increased uptake of amino acids as nutrients to satisfy their enhanced metabolic demands for rapid growth and proliferation. Furthermore, recent studies revealed the functional aspects of amino acids as signaling molecules. Especially, amino acids such as leucine are essential to activate mechanistic target of rapamycin complex 1 (mTORC1), a Ser/Thr-protein kinase complex that plays pivotal roles in regulating cell survival, growth, and proliferation and is often dysregulated in cancers [11–13]. L-type amino acid transporter 1 (LAT1; SLC7A5) [14], which preferentially transports large neutral amino acids, including most of the essential amino acids, is known to be upregulated in various types of cancers [14, 15]. The high expression level of LAT1 is associated with the poor prognosis of patients with multiple cancer types, including pancreatic and biliary tract cancers [16–18]. Due to its pathological function in cancer, LAT1 has been regarded as a rational target of molecularly targeted drugs.

Nanvuranlat (JPH203 or KYT-0353, abbreviated as NANV) is a LAT1-selective high-affinity inhibitor developed as the first-in-class anticancer agent [19, 20]. The anticancer effects of NANV have been well-proven preclinically against cancer cells from various organs in vitro [19, 21–30] and in vivo [19, 24, 25, 27, 31–33]. Consistent with the predominant contribution of LAT1 in supplying cancer cells with essential amino acids, including leucine, treatment with NANV reduces mTORC1 activity in cancer cells [21–24, 26, 27, 29–31, 33]. We have previously characterized the anticancer effects of NANV on pancreatic and biliary tract cancer cell lines [29, 30]. Inhibition of LAT1 with NANV suppressed the uptake of all the eight primary substrates of LAT1 into cancer cells and inhibited the mTORC1 pathway, resulting in a global suppression of protein synthesis [30]. Proteomics and phosphoproteomics revealed decreased phosphorylation of CDK1 and CDK2 [29] by NANV as possible regulators involved in the cell cycle arrest at the G0/G1 phase caused by the inhibition of LAT1 [25, 29, 33]. The first randomized phase II clinical trial of NANV monotherapy against pretreated, advanced, and refractory biliary tract cancers demonstrated a significant improvement in progression-free survival compared to placebo control (UMIN000034080) [34]. Notably, the safety profile of NANV was confirmed to be comparable to that of a placebo without developing any severe adverse events that lead to discontinuation, dose reduction, or death.

Because NANV targets the cancer cell-specific molecule LAT1, its combinational use with cytotoxic anticancer drugs may enhance the treatment efficacy while mitigating the risk of leading adverse effects and resistance [20]. We have previously shown that 2-aminobicyclo-(2,2,1)-heptane-2-carboxylic acid (BCH), a classical inhibitor of system L amino acid transporters including LAT1, in combination with the platinum drug cisplatin suppresses the growth of a head and neck squamous cell carcinoma cell line more strongly than by their single treatment [35]. However, due to its limited affinity and selectivity to LAT1, BCH was not further developed as an anticancer drug. It is still open to question whether the new anticancer drug NANV exhibits enhanced anticancer activity in combination with cytotoxic anticancer drugs or not.

In the present study, we first tested the combinations of NANV with seven distinct types of cytotoxic anticancer drugs to inhibit the growth of pancreatic cancer MIA PaCa-2 cells. NANV showed significantly enhanced growth inhibitory effects with all the tested drugs, where a relatively strong enhancement of growth inhibition was obtained in combination with GEM. The combined effects were also verified in multiple pancreatic and biliary tract cancer cell lines. We performed analyses of apoptosis, cell cycle, and phosphorylation of amino acid-related signaling proteins to elucidate the pharmacological mechanisms underlying the combined effects. Finally, the significant combined effects of GEM and NANV were verified in cancer cell spheroid cultures. This study reveals the potential of LAT1 inhibitor NANV as a concomitant drug with GEM to treat malignant pancreatic and biliary tract cancers.

## Methods

### Anticancer drugs

5-Fluorouracil (5-FU, Wako), 7-ethyl-10-hydroxycamptothecin (an active metabolite of irinotecan) (SN-38, Selleck), paclitaxel (TXL, Wako), and nanvuranlat (NANV, J-Pharma Co., Ltd.) were dissolved in DMSO. Gemcitabine hydrochloride (GEM, Wako), oxaliplatin (L-OHP, Wako), and doxorubicin hydrochloride (DXR, Wako) were dissolved in water. For all the tested drug concentrations, a constant volume of 333-fold drug stock solutions was added to the medium. Cyclophosphamide monohydrate (CPA, Wako) was directly dissolved in the medium.

### Cell culture

Pancreatic cancer HPAC (CRL-2119; ATCC), MIA PaCa-2 (JCRB0070; JCRB), PANC-1 (CRL-1469; ATCC), and SUIT-2 (JCRB1094; JCRB) cells and biliary tract cancer HuCCT1 (JCRB0425; JCRB), KKU-055 (JCRB1551; JCRB), KKU-100 (JCRB1568; JCRB), and KKU-213 (JCRB1557; JCRB) cells were cultured in RPMI-1640 supplemented with 10% FBS and 100 units/mL penicillin-100 µg/mL streptomycin. Cells were maintained in a humidified incubator at 37 °C supplied with 5% CO_2_.

### Cell growth assay

Cells were seeded at 1.0 × 10^3^ cells/well in 96-well plates (100 µL of medium/well). After 24 h of culture, the medium was replaced with a fresh medium containing the indicated concentrations of cytotoxic anticancer drug or NANV, or both. After 72 h of treatment, cell growth was measured by Cell Counting Kit-8 (Dojindo). Combined effects of drugs on cell growth were evaluated by the combination index (CI) based on the Bliss independence model using the following equation: CI = (E_A_+E_B _− E_A_E_B_)/E_AB,_ where E_A_ and E_B_ represent the observed growth inhibition by drug A and B, respectively, and E_AB_ by drug A combined with drug B. When CI is under, above, or equal to 1, the combined effects was judged as synergistic, antagonistic, or additive, respectively.

### Apoptosis assay

Cells were seeded at 3.0 × 10^4^ cells/well in 6 well plates (3 mL of medium/well). After 24 h, the medium was replaced with a fresh medium containing GEM or NANV, or both. After 72 h of incubation, apoptosis was analyzed by Muse™ Cell Analyzer (Millipore) using Muse™ Annexin V and Dead Cell kit. Annexin V and Dead Cell kit. Apoptotic rate (%) was expressed as the sum of the percentages of early (Annexin V-positive/7-AAD-negative) and late (Annexin V-positive/7-AAD-positive) apoptotic cells.

### Cell cycle analysis

Cells were seeded at 4.5 × 10^5^ cells/dish in 100 mm dishes containing 15 mL of medium and cultured for 48 h. Then, the cells were incubated for 24 h with a fresh medium containing GEM or NANV, or both. Cell cycle analysis was performed by Muse™ Cell Analyzer (Millipore) using Muse™ Cell Cycle kit.

### Western blot

Cells were seeded at 4.5 × 10^5^ cells/dish in 100 mm dishes containing 15 mL of medium and cultured for 48 h. Then, the cells were incubated for 24 h with a fresh medium containing GEM or NANV, or both. Western blot was performed as described previously [30]. Primary antibodies used are as follows: anti-β-actin (66009-1-Ig) from Proteintech; anti-phospho-Ser240/244-S6 ribosomal protein (5364), anti-S6 ribosomal protein (2217), anti-phospho-Ser51-eIF2α (3398), anti-eIF2α (5324), anti-phospho-Thr37/46-4EBP1 (2855), and anti-4EBP1 (9452) from Cell Signaling Technology.

### Spheroid culture

Cells were seeded in 96-well clear round bottom ultra-low attachment microplates (Corning, 7007) at 1.0 × 10^3^ cells in 100 µL/well of the medium. After centrifugation at 300×*g* for 10 min at 25 °C to sediment the cells, 100 µL of medium containing 10% (v/v) Matrigel (Falcon, 354230) was added to each well. Then the cells were cultured in a humidified incubator at 37 °C supplied with 5% CO_2_ to induce spheroid formation. After incubation for 72 h, 100 µL of the medium was replaced by 100 µL of a fresh medium containing either GEM or NANV, or both, at twice the final concentration (Day 0). On Day 3 and 5, 100 µL of the medium was replaced by 100 µL of a fresh medium containing the drug(s) at the indicated final concentrations. Bright-field images of spheroids were taken every 24 h by microscope (Leica, DMi1, MC120 HD). The projected area of spheroids was calculated using ImageJ software (NIH).

### Data reproducibility and statistical analysis

All the experiments were repeated at least twice to ensure the reproducibility of the results. Statistical analyses were performed with GraphPad Prism9 (GraphPad software). Differences were considered significant when *p*-values were < 0.05. *p < 0.05, **p < 0.01, ***p < 0.001, ****p < 0.0001, ns, not significant.

## Results

### Inhibition of pancreatic cancer MIA PaCa-2 cell growth by combinations of cytotoxic anticancer drugs and nanvuranlat

**Fig. 1:**
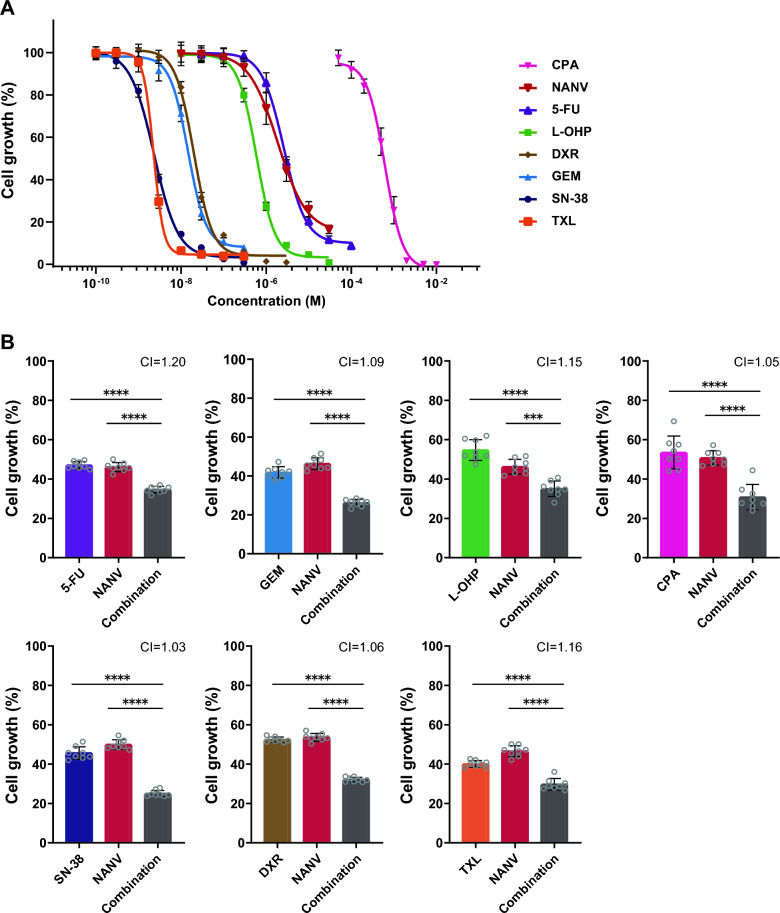
Inhibition of cell growth by single or combined treatment with cytotoxic anticancer drugs and nanvuranlat. MIA PaCa-2 cells were treated with drugs for 72 h. Cell growth was measured by Cell Counting Kit-8 (WST-8). Data were normalized by untreated controls and shown as mean ± SD (*n* = 8, technical replicates in a single experiment). **A** Concentration-dependent cell growth inhibition by single treatment with seven cytotoxic anticancer drugs and nanvuranlat (NANV). **B** Growth of MIA PaCa-2 cells treated with cytotoxic anticancer drugs or NANV, or both. Drugs were used at following concentrations: 5-FU, 3.5 µmol/L; GEM, 13 nmol/L; L-OHP, 2 µmol/L; CPA, 2.5 mmol/L; SN-38. 2.7 µmol/L; DXR, 20 nmol/L; TXL, 2.3 µmol/L; and NANV, 3 µmol/L. Statistical significance was evaluated by one-way ANOVA followed by Tukey's post-test. Combination indices (CI) were calculated based on the Bills independence model

We selected seven types of cytotoxic anticancer drugs with different mechanisms of action to test the antiproliferative effects in combination with NANV (Table 1). An active metabolite of irinotecan, SN-38, was used for the assay instead of irinotecan. The concentration-dependent inhibitory effects of each anticancer drug on cell growth were first confirmed in pancreatic cancer MIA PaCa-2 cells (Fig. 1A). The IC_50_ values of each drug under the experimental condition were determined to be as follows: 5-FU, 2.61 µmol/L; GEM, 14.70 nmol/L; L-OHP, 0.59 µmol/L; CPA, 0.62 mmol/L; SN-38, 2.33 nmol/L; DXR, 20.01 nmol/L; TXL, 2.27 nmol/L; and NANV, 1.87 µmol/L.

**Table 1:** Cytotoxic anticancer drugs used in this study

Classification	Drug
Antimetabolite (fluorinated pyrimidine, pyrimidine antagonist)	5-Fluorouracil (5-FU)
Antimetabolite (cytidine, pyrimidine antagonist)	Gemcitabine (GEM)
Platinum-based drug	Oxaliplatin (L-OHP)
Alkylating drug	Cyclophosphamide (CPA)
Topoisomerase-inhibiting drug (topoisomerase I)	Irinotecan (SN-38: metabolite of irinotecan)
Topoisomerase-inhibiting drug (topoisomerase II)	Doxorubicin (DXR)
Microtubule inhibitor	Paclitaxel (TXL)

We next investigated the growth inhibitory effects by combinations of cytotoxic anticancer drugs and NANV in MIA PaCa-2 cells. We set the experimental conditions so that a considerably high growth inhibition is achieved for evaluating the therapeutic potentials of the combinations. We also intended to select drug concentrations at which each of them exhibits discernible, but not saturated, pharmacological activities, allowing us to investigate the molecular mechanisms underlying the observed combination effects. Therefore, each drug was added to the medium at the concentration that inhibits cell growth by about 50% relative to untreated control cells. As shown in Fig. 1B, all the cytotoxic anticancer drugs combined with NANV suppressed cell growth significantly more strongly than their single treatment. Combination indices based on the Bliss independence model were nearly 1 for all the combinations, suggesting mostly additive but not synergistic effects [36, 37]. Relatively high enhancements of the cell growth inhibition were observed with GEM, CPA, SN-38, and DXR in combination with NANV. These results demonstrate the potential of LAT1 inhibitor NANV as a concomitant drug with various cytotoxic anticancer drugs.

### Inhibition of cell growth of multiple pancreatic and biliary tract cancer cell lines by the combination of gemcitabine and nanvuranlat

**Fig. 2:**
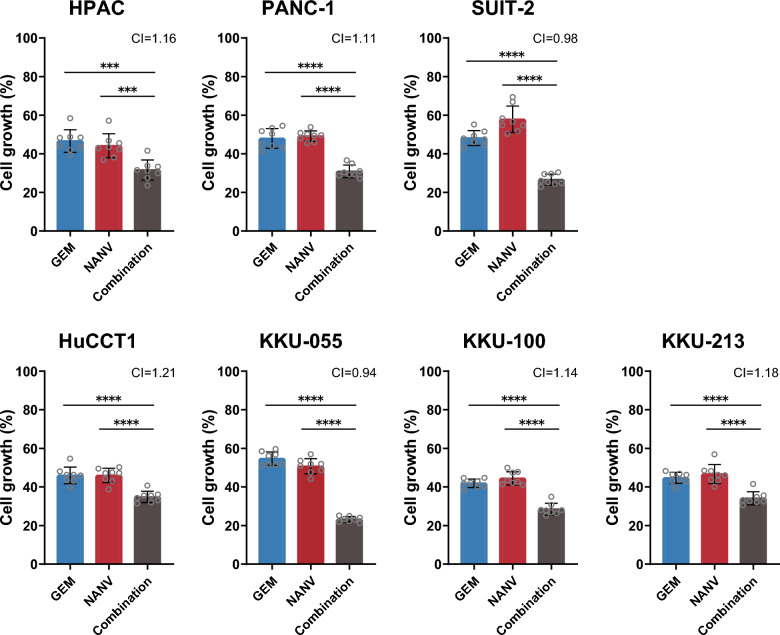
Inhibition of cell growth by single or combined treatment with gemcitabine and nanvuranlat in multiple pancreatic and biliary tract cancer cell lines. Pancreatic cancer (HPAC, PANC-1, and SUIT-2) and biliary tract cancer (HuCCT1, KKU-055, KKU-100, and KKU-213) cells were treated with GEM or NANV, or both, for 72 h. Cell growth was measured by Cell Counting Kit-8 (WST-8). Data were normalized by untreated controls and shown as mean ± SD (*n* = 8, technical replicates in a single experiment). Cells were treated with drugs at following concentrations: HPAC cells (GEM, 10 nmol/L; NANV, 6 µmol/L), PANC-1 cells (GEM, 150 nmol/L; NANV, 30 µmol/L), SUIT-2 cells (GEM, 3 nmol/L; NANV, 12 µmol/L), HuCCT1 cells (GEM, 20 nmol/L; NANV, 1.3 µmol/L), KKU-055 cells (GEM, 10 nmol/L; NANV, 0.9 µmol/L), KKU-100 cells (GEM, 6.5 nmol/L; NANV, 8 µmol/L), and KKU-213 cells (GEM, 40 nmol/L; NANV, 7 µmol/L). Statistical significance was evaluated by one-way ANOVA followed by Tukey's post-test. Combination indices (CI) were calculated based on the Bills independence model

GEM is the most commonly used anticancer agent in the current standard chemotherapy for advanced pancreatic and biliary tract cancers [4–6]. The favorable combinational effects of GEM and NANV on MIA PaCa-2 cell growth (Fig. 1B) prompted us to evaluate this combination in multiple pancreatic and biliary tract cancer cell lines. For this purpose, we selected three more pancreatic cancer cell lines (HPAC, PANC-1, and SUIT-2 cells) and four biliary tract cancer cell lines (HuCCT1, KKU-055, KKU-100, and KKU-213 cells). After confirming the concentration-dependent inhibition of cell growth by single treatments with GEM or NANV in each cell line (data not shown), the combined treatment was tested at the drug concentrations that inhibit the cell growth by about 50% relative to untreated control cells. The results revealed that the treatment of GEM in combination with NANV in all the tested cell lines exhibits significantly higher inhibitory effects on cell growth than every single treatment (Fig. 2).

In a previous study on head and neck squamous cell carcinoma cells, we reported the influence of the order of treatments with cisplatin and BCH, a canonical LAT1 inhibitor, on their combined effects. Pre-treatment of cells with cisplatin followed by BCH drastically enhanced the growth inhibitory effects compared to the reversed order of treatments [35]. Therefore, we evaluated the potential impact of the treatment order with GEM and NANV in MIA PaCa-2 and KKU-055 cells (Additional file 1: Fig. S1). The growth inhibition was significantly enhanced by sequential treatments of GEM and NANV compared to every single treatment in both cell lines, regardless of their order, while the growth inhibitory effects were inferior to the continuous and simultaneous treatment of GEM and NANV.

### Effects of the combination of gemcitabine and nanvuranlat on apoptosis

**Fig. 3:**
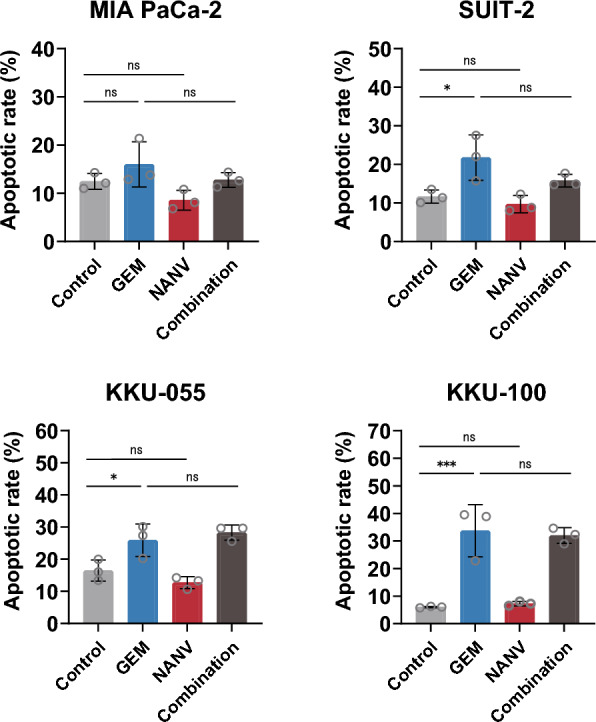
Induction of apoptosis by single or combined treatment with gemcitabine and nanvuranlat. Apoptosis induction was measured in MIA PaCa-2, SUIT-2, KKU-055, and KKU-100 cells treated with GEM or NANV, or both, for 72 h. Cells were treated with drugs at the following concentrations: MIA PaCa-2 cells (GEM, 13 nmol/L; NANV, 3 µmol/L), SUIT-2 cells (GEM, 3 nmol/L; NANV, 12 µmol/L), KKU-055 cells (GEM, 10 nmol/L; NANV, 0.9 µmol/L), and KKU-100 cells (GEM, 6.5 nmol/L; NANV, 8 µmol/L). Statistical significance was evaluated by one-way ANOVA followed by Tukey's post-test. Data were shown as mean ± SD (*n* = 3, biological replicates)

Apoptosis is associated with the anticancer activity of GEM [38]. To explore the molecular mechanisms underlying the combined effects of GEM and NANV on the growth of pancreatic and biliary tract cancer cells, we analyzed the induction of apoptosis in MIA PaCa-2, SUIT-2, KKU-055, and KKU-100 cells (Fig. 3). Experiments were performed using the same concentrations of GEM and NANV as that used for the cell growth inhibition assays. In SUIT-2, KKU-055, and KKU-100 cells, treatment with GEM alone significantly induced apoptosis compared to untreated control cells. Even though statistically insignificant, the same tendency was also observed in MIA PaCa-2 cells. In contrast, the single treatment of NANV did not induce apoptosis in any of the four tested cell lines. Apoptosis was not induced even when cells were treated with a higher concentration of NANV (30 µmol/L, data not shown), in line with its cytostatic anticancer activity. The co-treatment of GEM with NANV caused no increase in the apoptotic rate compared to the single treatment with GEM (Fig. 3).

### Effects of the combination of gemcitabine and nanvuranlat on cell cycle

**Fig. 4:**
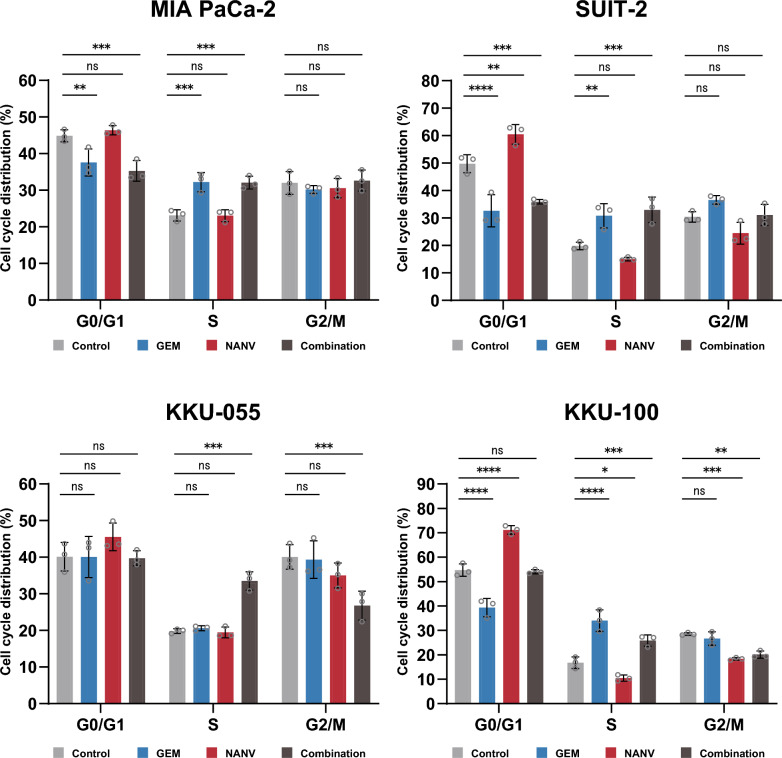
Effects of single or combined treatment with gemcitabine and nanvuranlat on cell cycle. MIA PaCa-2, SUIT-2, KKU-055, and KKU-100 cells were treated with GEM or NANV, or both, for 24 h and subjected to the cell cycle analysis. Cells were treated with drugs at the following concentrations: MIA PaCa-2 cells (GEM, 13 nmol/L; NANV, 3 µmol/L), SUIT-2 cells (GEM, 3 nmol/L; NANV, 12 µmol/L), KKU-055 cells (GEM, 10 nmol/L; NANV, 0.9 µmol/L), and KKU-100 cells (GEM, 6.5 nmol/L; NANV, 8 µmol/L). Data were shown as mean ± SD (*n* = 3, biological replicates). Statistical significance was evaluated by two-way ANOVA followed by Tukey's post-test

GEM and NANV are supposed to influence the cell cycle differently. GEM, as an antimetabolite, causes cell cycle arrest at the S phase [38]. Cell cycle arrest at the G0/G1 phase is involved in cell growth inhibition by NANV in biliary tract cancer cell lines [25, 29, 33]. Therefore, we next examined the effects of the combined use of GEM and NANV on the cell cycle (Fig. 4). The single treatment with NANV tended to increase the cells at the G0/G1 phase in all the cell lines, even though statistically not significant in MIA PaCa-2 and KKU-055 cells under this experimental condition. The proportion of the S phase cells was not altered in MIA PaCa-2, SUIT-2, and KKU-055 cells or slightly decreased in KKU-100 cells. Cells at the G2/M tend to be reduced by NANV in all the cell lines, although statistically significant only in KKU-100 cells.

The single treatment with GEM significantly increased the S phase cells and concomitantly decreased the G0/G1 phase cells in MIA PaCa-2, SUIT-2, and KKU-100 cells. In MIA PaCa-2 and SUIT-2 cells, the combined treatment of GEM and NANV increased cells at the S phase to the same extent as the GEM single treatment. In these cells, the G0/G1 phase cells were also decreased to the same extent as GEM single treatment. KKU-100 cells showed a significant increase in the proportion of the S phase cells when co-treated with GEM and NANV, while the extent was modest compared to GEM single treatment. Consistently, the decrease in the G0/G1 phase cells in KKU-100 cells was not so prominent as in MIA PaCa-2 and SUIT-2 cells. These results indicate that the combination of GEM and NANV tends to induce the cell cycle arrest at the S phase, relatively dominantly reflecting the pharmacological activity of GEM on the cell cycle (Fig. 4). Only in KKU-055 cells, the proportion of cells at the S phase was clearly increased by the combined treatment of GEM with NANV, but not by GEM alone. Interestingly, unlike other cell lines, the G0/G1 cells were at a similar level as the untreated control under the combined treatment, whereas the G2/M cells were significantly decreased in KKU-055 cells.

### Effects of the combination of gemcitabine and nanvuranlat on amino acid signaling

**Fig. 5:**
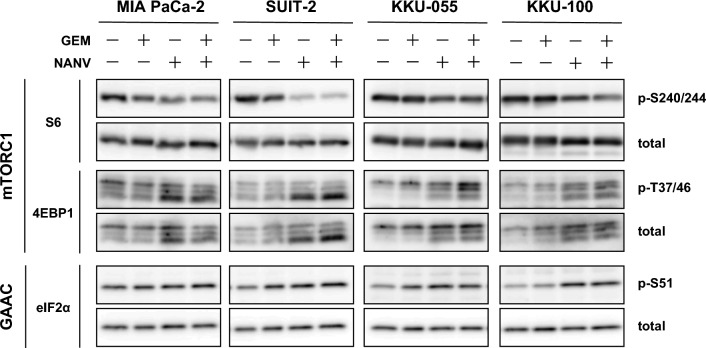
Effects of single or combined treatment with gemcitabine and nanvuranlat on amino acid signaling. MIA PaCa-2, SUIT-2, KKU-055, and KKU-100 cells were treated with GEM or NANV, or both, for 24 h, and analyzed by Western blot. Phosphorylated and total proteins of S6 ribosomal protein, 4EBP1, and eIF2α were detected. Cells were treated with drugs at the following concentrations: MIA PaCa-2 cells (GEM, 13 nmol/L; NANV, 3 µmol/L), SUIT-2 cells (GEM, 3 nmol/L; NANV 12 µmol/L), KKU-055 cells (GEM, 10 nmol/L; NANV 0.9 µmol/L), KKU-100 cells (GEM, 6.5 nmol/L; NANV, 8 µmol/L)

Amino acids transported by LAT1 are utilized as the material for protein synthesis and function as signaling molecules that activate mTORC1, a key regulator of cell metabolism and growth. One of the downstream directly regulated by mTORC1 is protein translation. As shown in Fig. 5, the single treatment with NANV decreased the phosphorylation of S6 ribosomal protein in all four cell lines, indicating the reduced activity of the mTORC1 pathway that positively regulates the translation in an amino acid-dependent manner. Another key protein in the mTORC1 pathway, 4EBP1, was detected as multiple bands, in which the slower mobility on gel represents the higher phosphorylation. NANV also suppressed the phosphorylation of 4EBP1, as demonstrated by the migration of bands to lower molecular weights. Another amino acid signaling pathway, general amino acid control (GAAC) pathway, is known to negatively regulate the translation initiation upon amino acid restriction. The phosphorylation of eIF2α was increased in all the cell lines except MIA PaCa-2 cells by NANV, indicating the increased activity of the GAAC pathway. All of these changes in the phosphorylation of molecules involved in amino acid signaling imply the suppression of translation by NANV.

The single treatment with GEM only limitedly influenced the phosphorylation of these amino acid signaling-related factors than that by NANV. The phosphorylation of S6 ribosomal protein was slightly decreased in MIA PaCa-2 cells, and that of eIF2α was slightly increased in SUIT-2 and KKU-055 cells. In all the cell lines, the combined treatment of NANV with GEM induced similar levels of the change in phosphorylation as the treatment with NANV alone (Fig. 5). These results suggest that co-treatment with GEM does not enhance the influence of NANV on mTORC1 and GAAC pathways, and subsequent protein synthesis.

### Combined growth inhibitory effects of gemcitabine and nanvuranlat in the spheroid culture of pancreatic and biliary tract cancer cell lines

**Fig. 6:**
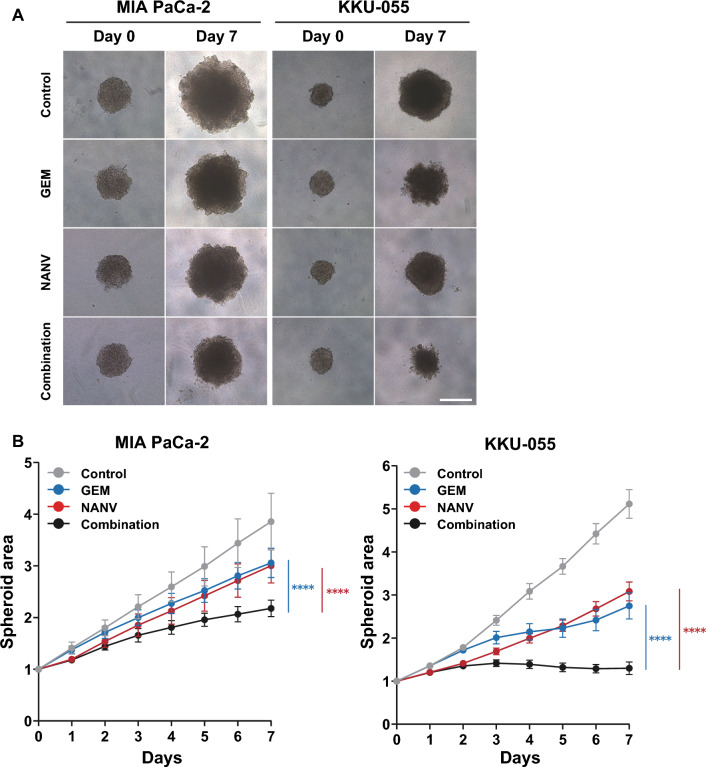
Inhibition of spheroid growth by single or combined treatment with gemcitabine and nanvuranlat. Spheroids of MIA PaCa-2 and KKU-055 cells were treated with GEM or NANV, or both, at the following concentrations: MIA PaCa-2 cells (GEM, 15 nmol/L; NANV, 30 µmol/L) and KKU-055 cells (GEM, 12.5 nmol/L, NANV, 1 µmol/L). Drug treatment was started on Day 0. The half-volume medium exchange was done on Day 3 and Day 5. **A** Representative bright-field images of spheroids on Day 0 and Day 7. Scale bar: 500 μm. **B** Quantification of the spheroid growth. The projected area size of each spheroid on each day was calculated from the bright-field image and normalized by Day 0. Data were shown as mean ± SD (*n* = 10, technical replicates in a single experiment). Statistical significance was evaluated by two-way ANOVA followed by Tukey's post-test

All the above results in two-dimensional cancer cell cultures suggested a promising therapeutic potential of NANV as a concomitant drug with GEM against pancreatic and biliary tract cancers. To obtain further support for this possibility in an assay that more accurately recapitulates the actual tumor tissues, we performed growth inhibition experiments using spheroid cultures (Fig. 6). Spheroids were constructed from MIA PaCa-2 and KKU-055 cells. The single treatments with GEM or NANV significantly but moderately suppressed the growth of spheroids in both cell lines. Notably, by co-treating spheroids with GEM and NANV, the suppression of spheroid growth was profoundly enhanced compared to their single treatment. As shown in Fig. 6B, the single treatment by NANV significantly suppressed the spheroid growth as early as 24 h after starting the treatment, while GEM single treatment did not until 96 h (MIA PaCa-2 cells) or 72 h (KKU-055 cells). These observations are consistent with the results of experiments in two-dimensional cultures, where the cell growth inhibition by NANV was detectable within 24 h and preceded that by GEM that required longer than 24 h to induce apoptosis (confirmed at 48 and 72 h, data not shown). Under the combinational treatment with GEM and NANV, the growth inhibition until 48 h after starting the treatment may mainly reflect the effects of NANV. The sustained inhibition of spheroid growth in longer incubation time seems to be attributed to the additive effects of GEM and NANV.

## Discussion

In this study, we first examined the combinations of seven distinct types of cytotoxic anticancer drugs with an amino acid transporter LAT1 inhibitor, nanvuranlat (NANV; JPH203 or KYT-0353), on the growth of pancreatic cancer MIA PaCa-2 cells (Fig. 1B). All the tested combinations showed significantly enhanced growth inhibitory effects compared to their single treatments. The combined effects were suggested to be primarily additive under the current experimental conditions. Whereas we previously reported the combination effects of BCH and cisplatin against head and neck cancer cells [35], BCH is a compound with a broad specificity over system L amino acid transporters [39]. The obtained anticancer effects thus cannot be specifically attributed to the inhibition of LAT1. For the first time, this study revealed the combined growth inhibitory effects specifically obtained by LAT1 inhibition with various types of cytotoxic anticancer drugs. Considering that NANV has demonstrated anticancer effects against cancer cells derived from various organs in preclinical studies [19, 21–30], these results suggest the potential of NANV for broad clinical applications against multiple types of cancers in combination with cytotoxic anticancer drugs. Among the tested drugs, NANV exhibited relatively high combined effects with GEM, CPA, SN-38, and DXR. Therefore, we selected combining GEM with NANV for further evaluation against pancreatic and biliary tract cancer cells because GEM-based drug therapies are standard treatments for these refractory cancer types but remain ineffective [4–6]. The favorable outcomes of the first phase II clinical trial of NANV monotherapy in pretreated, advanced, and refractory biliary tract cancers encouraged us to pursue this possibility (UMIN000034080) [34]. As a result, significant enhancement of the growth inhibitory effects by combining GEM and NANV was demonstrated in all the tested pancreatic and biliary tract cancer cell lines (four cell lines for each cancer type) (Figs. 1B and 2). The combination effects were observed not only in two-dimensional cultures but also in spheroid cultures of cancer cells (Fig. 6).

To elucidate the molecular basis for the combined effects of GEM and NANV, we performed analyses of the cell cycle, apoptosis, and amino acid-related signaling. The obtained overall results revealed no apparent enhancement in the pharmacological activities of each drug under the current experimental conditions. Consistent with the previous reports [38], the single treatment with GEM induced cell cycle arrest at the S phase and apoptosis. NANV alone induced cell cycle arrest at the G0/G1 phase and did not induce apoptosis, as shown in previous research [25, 29, 33]. The combination of GEM and NANV caused the cell cycle arrest at the S phase and induced apoptosis to similar levels as GEM alone in MIA PaCa-2, SUIT-2, and KKU-100 cells (Figs. 3 and 4). Therefore, GEM is supposed to influence the cell cycle and apoptosis more dominantly than NANV in their combination. An exceptional observation was made in the cell cycle analysis of the KKU-055 cell. The proportion of cells at the S phase was increased by combining GEM with NANV, but not by GEM alone. Notably, the G0/G1 cells were at a similar level as the untreated control under the combined treatment, whereas the G2/M cells were significantly decreased in KKU-055 cells. Although the details remain to be elucidated, these observations suggest that the increase of S phase cells in KKU-055 cells by the combined treatment with GEM and NANV cannot be simply interpreted as the enhanced activity of GEM that induces the S phase arrest by decreasing cells at the G0/G1 phase. Consistently, NANV did not potentiate the apoptosis-inducing activity of GEM in KKU-055 cells. Treatments with NANV altered the phosphorylation levels of proteins in amino acid-related signaling pathways to similar levels, irrespectively to the presence or the absence of GEM (Fig. 5). The identified changes in the phosphorylation in mTORC1 and GAAC pathways suggest the suppression of protein synthesis, representing the pharmacological activity of NANV without noticeable augmentation by GEM. These results indicate that GEM and NANV mostly independently exert their anticancer activities even in combination.

This study investigated the combination of GEM and NANV at a single dose set. We focused on revealing the general molecular mechanisms underlying the combination effects using multiple pancreatic and biliary tract cell lines. Conversely, the concentrations and ratio of the two drugs remain to be optimized to attain the best combination effects. Furthermore, we adopted the Bliss independence model [36] to evaluate the drug combination effects because the mechanisms of action of cytostatic NANV and cytotoxic anticancer drugs are regarded as primarily independent. However, all the available reference models still present some limitations and do not perfectly fit the actual experimental conditions [37, 40]. The analyses of GEM and NANV in this study implied that their detailed pharmacological activities are not completely independent and partially interfere with each other, as exemplified in their effects on the cell cycle, where the effects of NANV to induce the G0/G1 arrest was generally masked when combined with GEM (Fig. 4). The cooperative use of multiple theoretical and experimental methods [37, 40] thus would be important to reinforce the significance of our findings in future studies.

Nevertheless, because NANV is the first-in-class anticancer drug targeting LAT1, the discovery of GEM as a preferable combination partner holds significant implications for its future clinical development. The findings of this study may contribute to developing novel therapeutic strategies with GEM, which is currently widely used for pancreatic and bile duct cancers. Notably, cancer cell-specific cytostatic anticancer activities of NANV may pave the way to circumvent the problems of adverse effects and drug resistance posed by GEM (and other cytotoxic anticancer drugs). Significant combination effects of a mTORC1 inhibitor, temsirolimus, and GEM have been reported previously in an animal model of pancreatic cancer [41], while failed to show clinical efficacy in the first phase I/II study [42]. It has also been reported that another mTORC1 inhibitor, everolimus, shows more pronounced antiproliferative effects against GEM-resistant pancreatic cancer cells than against GEM-sensitive pancreatic cancer cells [43] and exhibits synergistic antiproliferative effects with GEM against biliary tract cancer cells [44]. In addition to inhibiting mTORC1 by blocking the essential input of amino acid signals, NANV induces the depletion of amino acids as biosynthetic materials and suppresses the global translation in cancer cells [30]. Thus, combining NANV with GEM may exhibit robust and multifaceted anticancer effects based on such broad pharmacological activities. A particularly tempting speculation in this regard would be that NANV, co-administrated with GEM, inhibits cancer cell growth by generally suppressing protein synthesis and prevents the acquisition of drug resistance by abolishing the expression of proteins involved in the resistance to GEM [38, 45]. Future studies should also investigate such possible mechanistic convergence in their anticancer activities that may lead to better combination effects.

## Conclusions

This study provides the primary evidence for the combinational effects of gemcitabine with a novel molecularly targeted drug, nanvuranlat, that may propose effective treatments for malignant pancreatic and bile duct cancers. The two drugs, when combined, additively suppressed the growth of cancer cells by exhibiting their pharmacological activities largely independently under the tested conditions. To further explore the in vivo relevance of our findings, detailed conditions for drug treatments, especially the concentrations and ratio of the two drugs, need to be further optimized to accomplish the best combination effects. Validation of the combination effects based on the two or more mutually compensative evaluation methods will also be particularly important. Results of such future studies will provide valuable information to extrapolate and enhance the combined effects of gemcitabine and nanvuranlat in in vivo animal models and clinical settings.

## Supplementary Information

**Additional file 1: Figure S1.** Combination effects of gemcitabine and nanvuranlat on cell growth by sequential treatments. **A** The treatment schedule of single or simultaneous treatments with gemcitabine and nanvuranlat. According to the schedule, MIA PaCa-2 and KKU-055 cells were treated with GEM or NANV, or both. After 36 h of the first treatment, the medium was removed, washed once with 100 μL of the medium, and replaced with fresh medium containing drugs for a further 36 h of treatment. **B** Cell growth inhibition was analyzed by Cell Counting Kit-8 after 72 h of the treatments. Cells were treated with drugs at the following concentrations: MIA PaCa-2 (GEM; 13 nmol/L, NANV; 3 μmol/L), KKU-055 (GEM; 10 nmol/L, NANV; 0.9 μmol/L). Data were normalized for non-treated controls and shown as mean ± SEM (*n* =8). Statistical significance was evaluated by one-way ANOVA followed by Tukey's post-test.

## Data Availability

All the data analyzed and presented in this study are available from the authors upon reasonable request.

## Abbreviations

GEM: Gemcitabine
mTORC1: Mechanistic target of rapamycin complex 1
LAT1: L-type amino acid transporter 1
NANV: Nanvuranlat
BCH: 2-Aminobicyclo-(2,2,1)-heptane-2-carboxylic acid
5-FU: 5-Fluorouracil
SN-38: 7-Ethyl-10-hydroxycamptothecin
TXL: Paclitaxel
L-OHP: Oxaliplatin
DXR: Doxorubicin
CPA: Cyclophosphamide
GAAC: General amino acid control
