# Supplementary material for: Combination effects of amino acid transporter LAT1 inhibitor nanvuranlat and cytotoxic anticancer drug gemcitabine on pancreatic and biliary tract cancer cells
Source: Cancer Cell Int. 2023 Jun 15;23:116. doi: 10.1186/s12935-023-02957-z (PMC10268460; doi:10.1186/s12935-023-02957-z)
Supplement: Supplementary file 1 — Additional file 1: Figure S1. Combination effects of gemcitabine and nanvuranlat on cell growth by sequential treatments. A The treatment schedule of single or simultaneous treatments with gemcitabine and nanvuranlat. According to the schedule, MIA PaCa-2 and KKU-055 cells were treated with GEM or NANV, or both. After 36 h of the first treatment, the medium was removed, washed once with 100 μL of the medium, and replaced with fresh medium containing drugs for a further 36 h of treatment. B Cell growth inhibition was analyzed by Cell Counting Kit-8 after 72 h of the treatments. Cells were treated with drugs at the following concentrations: MIA PaCa-2 (GEM; 13 nmol/L, NANV; 3 μmol/L), KKU-055 (GEM; 10 nmol/L, NANV; 0.9 μmol/L). Data were normalized for non-treated controls and shown as mean ± SEM (n =8). Statistical significance was evaluated by one-way ANOVA followed by Tukey's post-test. [file 12935_2023_2957_MOESM1_ESM.pdf]

Additional file 1

Combination effects of amino acid transporter LAT1 inhibitor nanvuranlat and cytotoxic anticancer drug gemcitabine on pancreatic and biliary tract cancer cells

Kou Nishikubo[1], Ryuichi Ohgaki[1, 2], Xingming Liu[1], Hiroki Okanishi[1], Minhui Xu[1], Hitoshi Endou[3] and Yoshikatsu Kanai[1,2]

[1]Department of Bio-system Pharmacology, Graduate School of Medicine, Osaka University, Osaka, Japan

[2]Integrated Frontier Research for Medical Science Division, Institute for Open and Transdisciplinary Research Initiatives (OTRI), Osaka University, Osaka, Japan

[3]J-Pharma Co., Ltd., Yokohama, Japan

Correspondence

Yoshikatsu Kanai and Ryuichi Ohgaki, 2-2 Yamadaoka, Suita, Osaka 565-0871, Japan.

Email: ykanai@pharma1.med.osaka-u.ac.jp, ohgaki@pharma1.med.osaka-u.ac.jp

TEL: 06-6879-3521

FAX: 06-6879-3529

Additional file 1

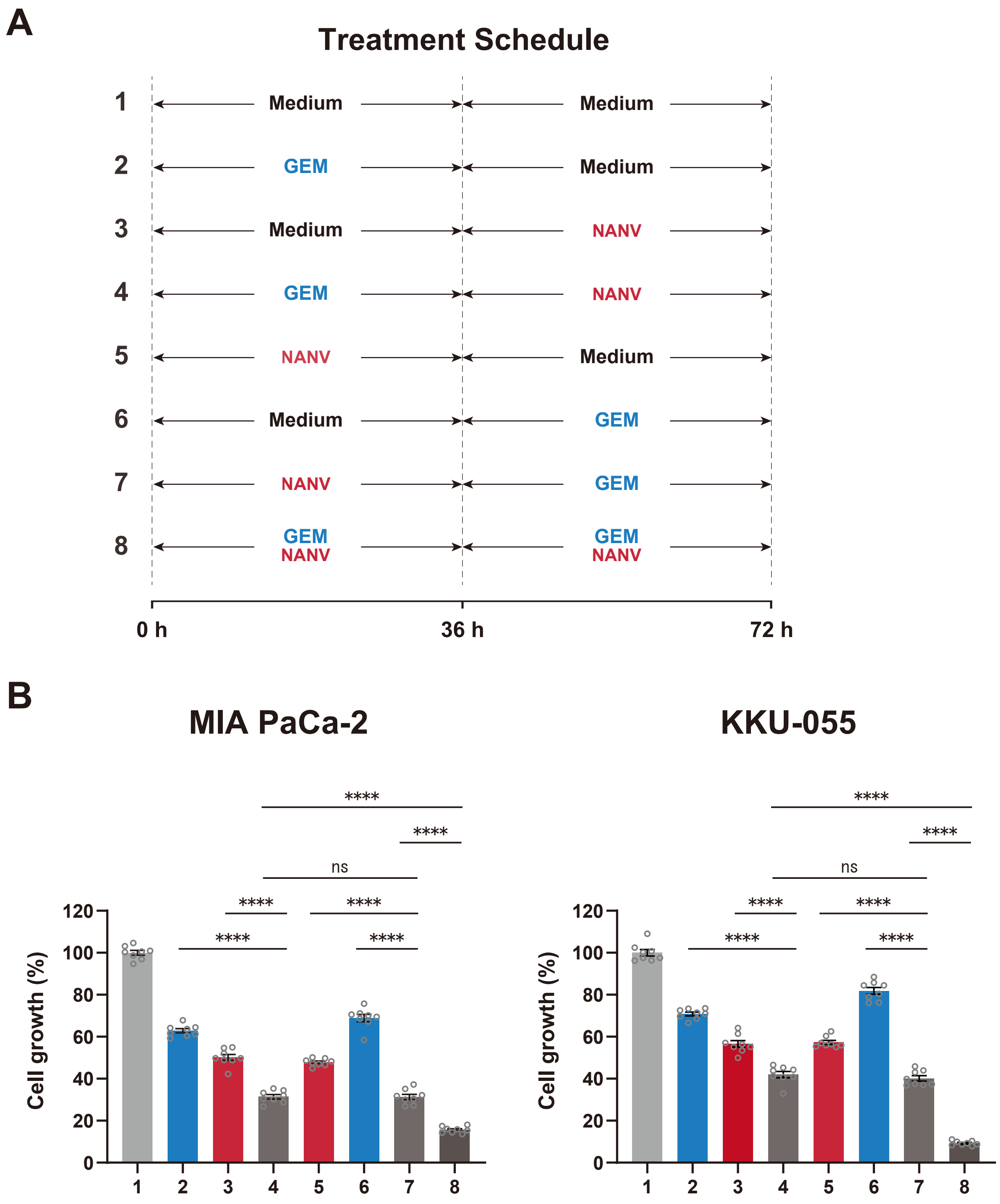

Figure S1

Combination effects of gemcitabine and nanvuranlat on cell growth by sequential treatments. **A** The treatment schedule of single or simultaneous treatments with gemcitabine (GEM) and nanvuranlat (NANV). According to the schedule, MIA PaCa-2 and KKU-055 cells were treated with GEM or NANV, or both. After 36 h of the first treatment, the medium was removed, washed once with 100 μL of the medium, and replaced with fresh medium containing drugs for a further 36 h of treatment (72 h in total). **B** Cell growth inhibition was analyzed by Cell Counting Kit-8 after 72 h of the treatments. Cells were treated with drugs at the following concentrations: MIA PaCa-2 (GEM; 13 nmol/L, NANV; 3 μmol/L), KKU-055 (GEM; 10 nmol/L, NANV; 0.9 μmol/L). Data were normalized for non-treated controls and shown as mean ± SEM (n = 8). Statistical significance was evaluated by one-way ANOVA followed by Tukey's post-test.
